# Adequacy of prenatal care among women living with human immunodeficiency virus: a population-based study

**DOI:** 10.1186/s12889-015-1842-y

**Published:** 2015-05-29

**Authors:** Ryan Ng, Erin M Macdonald, Mona R Loutfy, Mark H Yudin, Janet Raboud, Khatundi-Irene Masinde, Ahmed M Bayoumi, Wangari E Tharao, Jason Brophy, Richard H Glazier, Tony Antoniou

**Affiliations:** Institute for Clinical Evaluative Sciences, Toronto, Ontario Canada; Institute of Health Policy, Management and Evaluation, University of Toronto, Toronto, Ontario Canada; Department of Medicine, University of Toronto, Toronto, Ontario Canada; Women’s College Research Institute, Women’s College Hospital, Toronto, Ontario Canada; Li Ka Shing Knowledge Institute, St. Michael’s Hospital, Toronto, Ontario Canada; Centre for Research on Inner City Health, St. Michael’s Hospital, Toronto, Ontario Canada; Department of Obstetrics and Gynecology, St. Michael’s Hospital and University of Toronto, Toronto, Ontario Canada; Toronto General Research Institute, University Health Network, Toronto, Ontario Canada; Dalla Lana School of Public Health, University of Toronto, Toronto, Ontario Canada; Women’s Health in Women’s Hands Community Health Centre, Toronto, Ontario Canada; Children’s Hospital of Eastern Ontario and University of Ottawa, Ottawa, Ontario Canada; Department of Family and Community Medicine, St. Michael’s Hospital and University of Toronto, Toronto, Ontario Canada

**Keywords:** Prenatal care, HIV, Disparity, Population-based, Immigrant

## Abstract

**Background:**

Prenatal care reduces perinatal morbidity. However, there are no population-based studies examining the adequacy of prenatal care among women living with HIV. Accordingly, we compared the prevalence of adequate prenatal care among women living with and without HIV infection in Ontario, Canada.

**Methods:**

Using administrative data in a universal single-payer setting, we determined the proportions of women initiating care in the first trimester and receiving adequate prenatal care according to the Revised-Graduated Prenatal Care Utilization Index . We also determined the proportion of women with HIV receiving adequate prenatal care by immigration status. We used generalized estimating equations with a logit link function to derive adjusted odds ratios (aORs) and 95 % confidence intervals (CI) for all analyses.

**Results:**

Between April 1, 2002 and March 31, 2011, a total of 1,132,135 pregnancies were available for analysis, of which 634 (0.06 %) were among women living with HIV. Following multivariable adjustment, women living with HIV were less likely to receive adequate prenatal care (36.1 % versus 43.3 %; aOR 0.74, 95 % CI 0.62 to 0.88) or initiate prenatal care in the first trimester (50.8 % versus 70.0 %; aOR 0.51, 95 % CI 0.43 to 0.60) than women without HIV. Among women with HIV, recent (i.e. ≤ 5 years) immigrants from Africa and the Caribbean were less likely to receive adequate prenatal care (25.5 % versus 38.5 %; adjusted odds ratio 0.51; 95 % CI, 0.32 to 0.81) than Canadian-born women.

**Conclusion:**

Despite universal health care, disparities exist in the receipt of adequate prenatal care between women living with and without HIV. Interventions are required to ensure that women with HIV receive timely and adequate prenatal care.

**Electronic supplementary material:**

The online version of this article (doi:10.1186/s12889-015-1842-y) contains supplementary material, which is available to authorized users.

## Background

Prenatal care is among the most widely used preventive health care service in developed countries [1].The Society of Obstetricians and Gynaecologists of Canada recommends that prenatal care visits are scheduled every 4 to 6 weeks during early pregnancy, every 2 to 3 weeks after 30 weeks' gestation, and every 1 to 2 weeks after 36 weeks' gestation [2]. Early initiation of and retention in prenatal care provides expectant mothers access to a variety of medical, nutritional and educational interventions directed towards optimizing infant health and has been associated with reduced risks of adverse neonatal and maternal outcomes [3–6]. However, despite widespread endorsement by national medical societies [7, 8], several studies have suggested that disparities exist with respect to the receipt of adequate prenatal care, commonly defined as the initiation of care in the first trimester or according to a prenatal care utilization index that considers the number and periodicity of prenatal care visits in relation to the gestational age of the newborn at delivery [9]. Specifically, higher parity, belonging to a minority ethnic group and low socioeconomic status have consistently been associated with inadequate utilization of prenatal care services [10–12]. In addition, women who have immigrated to Western countries are less likely to initiate prenatal care in the first trimester and attend prenatal appointments relative to non-immigrant women for reasons that include poor language proficiency, community isolation and institutionalized racism [13–18]. In the context of HIV-infection, these factors intersect with multiple forms of perceived and enacted stigma within the health care system to further undermine engagement with health services and preconception counselling [19–21]. Notably, several studies have found that approximately 50 % of women with HIV do not receive reproductive counselling from their health care providers [22–24]. Furthermore, previous research has shown that 54 % of pregnancies among a cohort of women living with HIV in Ontario were unplanned, compared with 30 % in the general Ontario population [25]. These findings are concerning because unintended pregnancies delay the initiation of prenatal care, the timely initiation of which has been shown to reduce the risks of preterm births and low birth weight infants among women with HIV [26, 27].

Despite these data, little is known about the impact of maternal HIV infection on the use of prenatal care services. Studies conducted during the early years of the epidemic found that up to two-thirds of women living with HIV in the United States did not receive adequate prenatal care as assessed with a prenatal care utilization index, but inferences from these studies are limited by a lack of generalizability to contemporary HIV care [28]. To our knowledge, there are no population-based studies examining the adequacy of prenatal care for women with HIV in a setting of universal health insurance. Accordingly, we studied the adequacy of prenatal care in women living with HIV relative to women not living with HIV in Ontario, Canada. Because women originally from Africa and the Caribbean account for an increasing proportion of women living with HIV who are having children in Ontario (26.7 % in 2002/2003 to 51.6 % in 2009/10), we also examined the adequacy of prenatal care among women with HIV originally from these regions [29]. In light of research describing stigma and factors related to immigration as barriers to accessing care, we hypothesized that women with HIV would be less likely to receive adequate prenatal care than women without HIV. Similarly, we hypothesized that women with HIV who were originally from Africa and the Caribbean would be less likely to receive adequate prenatal care than non-immigrant women.

## Methods

### Setting

We conducted a population-based study of pregnancies among women living with and without HIV in Ontario between April 1, 2002 and March 31, 2011. Ontario has a universal single-payer, government administered health care system. Accordingly, all permanent residents are eligible to receive publicly funded physician and hospital care, including prenatal care, without copayments or deductibles. This project was approved by the Research Ethics Board of the Sunnybrook Health Sciences Centre, Toronto, Ontario.

### Data sources

We used Ontario’s administrative health databases, which were held securely in linkable files without any direct personal identifiers, and analyzed at the Institute for Clinical Evaluative Sciences. Specifically, we identified all pregnancies among Ontario women between the ages of 18 and 49 during the study period using the MOMBABY database, which deterministically links the Canadian Institute for Health Information Discharge Abstract Database inpatient admission records of all mothers and their newborn infants from 2002/03 onward. From within this cohort, we identified births to women living with HIV using the Ontario HIV Database, an administrative data registry of Ontario residents with diagnosed HIV infection which was generated using a previously validated case-finding algorithm [30]. We obtained demographic information from the Registered Persons Database, a registry of all Ontario residents eligible for provincial health insurance. We used the Ontario Health Insurance Plan (OHIP) database to identify physician claims for prenatal visits and obtained hospitalization data from the Canadian Institute for Health Information Discharge Abstract Database. Finally, we used the Citizenship and Immigration Canada Database to identify women who had immigrated to Ontario and their country of origin. These databases were linked in an anonymous fashion using encrypted health card numbers, and are routinely used for population-based research examining health care delivery and health outcomes, including pregnancy outcomes [31, 32].

### Outcome

The primary outcome was the receipt of adequate prenatal care, determined using the Revised-Graduated Prenatal Care Utilization Index (R-GINDEX) [33]. The R-GINDEX, which is based on recommendations of the American College of Obstetricians and Gynecologists, classifies the adequacy of care into one of six categories ( inadequate, adequate, intermediate, intensive, no care or missing) based on the number of prenatal care visits, the gestational age of the newborn at birth and the date prenatal care was initiated. For example, women who give birth at 40 weeks gestation and who initiate care in the first trimester would be classified as having received adequate, intermediate or inadequate care if they received between 13 and 16 visits, 8 and 12 visits or fewer than 8 visits, respectively (see Additional file 1: Table S1 for examples of R-GINDEX categories for 28-, 32-, 36- and 40-week deliveries). We determined the gestational age of the newborn from the MOMBABY database and used the OHIP database to identify all prenatal office visits to primary care physicians and obstetrician/gynecologists. We did not include visits where only screening procedures such as amniocentesis or ultrasounds were performed. We classified women in the ‘adequate’ and ‘intensive’ categories as receiving adequate prenatal care, and women in the ‘inadequate’, ‘no care’ and ‘intermediate’ categories as receiving inadequate prenatal care. We also determined the proportions of women living with and without HIV who initiated care in the first trimester, because this is a traditionally used measure of prenatal care adequacy [34–36]. Women with missing prenatal care (i.e. missing gestational age) according to the R-GINDEX were not included in the analyses.

### Statistical analyses

We compared baseline characteristics of pregnancies to mothers living with and without HIV using one-way analysis of variance for continuous variables, Cochrane-Armitage tests for ordinal variables and chi-square tests for categorical variables. In separate analyses, we compared the proportions of women living with and without HIV who received adequate prenatal care according to the R-GINDEX and who initiated prenatal care in the first trimester using multivariable general estimating equations with a logit link function and an exchangeable correlation structure to account for multiple pregnancies from the same woman during the follow-up period. We adjusted models for variables known to influence the use of prenatal care services, including age, parity, multiple versus singleton birth, co-morbid disease, immigration status, and socioeconomic status. We categorized time since immigration to Ontario as recent (i.e. ≤ 5 years) or non-recent (i.e. >5 years). We used Johns Hopkins Aggregated Diagnosis Groups, which range from 0 (no diagnosis groups) to a maximum of 32 distinct diagnosis groups, to adjust for differences in comorbidity between women with and without HIV [37]. We used the Ontario Marginalization Index as a measure of maternal socioeconomic status [38]. Specifically, we derived quintiles of neighborhood material deprivation and residential instability using postal code data from the Registered Persons Database and the 2001 census of Canada. For example, an individual who lives in a neighborhood in the fifth quintile on the material deprivation scale resides in one of the 20 % most deprived areas in Ontario. Residential instability includes seven census measures: percentage living alone, percentage youth 5–15 years, persons per dwelling, percentage living in apartment buildings, percentage married, percentage home ownership, and percentage moving within the last 5 years [38]. Similarly, material deprivation includes six census measures, expressed as proportions: aged ≥20 years without high school graduation, lone parent families, population receiving government transfer payments, aged ≥15 and unemployed, living below the low income cut-off, and homes needing major repairs [38]. In separate models, we examined whether region of origin and time since immigration were associated with receiving adequate prenatal care only among women living with HIV. All analyses were performed using SAS version 9.3 (Cary, NC).

## Results

We identified 1,133,522 pregnancies between April1, 2002 and March 31, 2012. After excluding 1,387 (0.1 %) pregnancies for which the R-GINDEX could not be determined (all to HIV-negative women), 1,132,135 pregnancies were available for analysis, of which 634 (0.06 %) were among women living with HIV. Relative to women without HIV, women living with HIV were more likely to be immigrants (48.1 % vs. 25.9 %; p < 0.001) and live in neighborhoods that were the most deprived (41.1 % vs. 16.2 %; p < 0.001) and with the greatest residential instability (39.9 % vs. 17.9 %; p < 0.001) (Table 1). Women with HIV were also more likely to have a pregnancy resulting in multiple births (3.0 % vs. 1.8 %; p = 0.02) and a greater co-morbidity burden, as demonstrated by the median number of Aggregated Diagnosis Groups in the preceding year [6 (interquartile range 5.0 to 9.0) vs. 4.0 (interquartile range 3.0 to 6.0); p < 0.001] (Table 1).

**Table 1:** Baseline characteristics of pregnancies according to HIV status

Characteristic	HIV	Non-HIV	p-value
	(n = 634)	(n = 1,131,501)	
Mean age ± SD (years)	30.8 ± 5.2	30.1 ± 5.2	0.001
18 to 34 years	470 (74.1 %)	894,604 (79.1 %)	0.002
35 to 49 years	164 (25.9 %)	236,897 (20.9 %)	
Aggregated Diagnosis Groups			
Median (IQR)	6.0 (5.0 – 9.0)	4.0 (3.0 – 6.0)	< 0.001
0 to 5	238 (37.5 %)	786,857 (69.5 %)	< 0.001
6 to 10	331 (52.2 %)	330,199 (29.2 %)	
11 or more	65 (10.3 %)	14,445 (1.3 %)	
Immigration Status, No. (%)			< 0.001
Non-immigrant	329 (51.9 %)	839,572 (74.2 %)	
Non-recent immigrant, Africa or Caribbean	97 (15.3 %)	23,788 (2.1 %)	
Non-recent immigrant, other world regions	28 (4.4 %)	108,338 (9.6 %)	
Recent immigrant, Africa or Caribbean	157 (24.8 %)	15,393 (1.4 %)	
Recent immigrant, other world regions	23 (3.6 %)	144,410 (12.8 %)	
Material Deprivation Income Quintile, No. (%)			< 0.001
1 (lowest)	68 (10.7 %)	296,497 (26.2 %)	
2	72 (11.4 %)	232,786 (20.6 %)	
3	98 (15.5 %)	213,374 (18.9 %)	
4	117 (18.5 %)	190,740 (16.9 %)	
5	261 (41.2 %)	183,633 (16.2 %)	
Residential Instability Quintile, No. (%)			< 0.001
1 (lowest)	77 (12.1 %)	303,196 (26.8 %)	
2	72 (11.4 %)	228,544 (20.2 %)	
3	69 (10.9 %)	168,253 (14.9 %)	
4	145 (22.9 %)	214,623 (19.0 %)	
5	253 (39.9 %)	202,414 (17.9 %)	
Multiple birth	19 (3.0 %)	19,833 (1.8 %)	0.02
Median (IQR) gestational age (weeks)	38 (37 – 40)	39 (38 – 40)	< 0.001

SD, standard deviation; IQR, interquartile range

The median number of prenatal care visits by women living with and without HIV was 11.0 (interquartile range 8.0 to 13.0) and 11.0 (interquartile range 9.0 to 13.0), respectively (p < 0.001). However, the proportion of women initiating prenatal care in the first trimester (50.8 % vs. 70.0 %; p < 0.001) was significantly lower among women living with HIV. When classified using the R-GINDEX, adequate prenatal care was received by 36.1 % and 43.3 % of women living with and without HIV, respectively (p < 0.001).

Following multivariable adjustment, women living with HIV were less likely to receive adequate prenatal care [adjusted odds ratio (aOR) 0.74; 95 % confidence interval (CI) 0.62 to 0.88] or initiate prenatal care in the first trimester (aOR 0.51; 95 % CI 0.43 to 0.60) than women living without HIV (Tables 2 and 3). The odds of receiving adequate prenatal care and care in the first trimester also decreased with increasing neighborhood deprivation (Tables 2 and 3). Among women with HIV, recent (i.e. ≤ 5 years) immigrants from Africa and the Caribbean were less likely to receive adequate prenatal care (25.5 % vs. 38.5 %; aOR 0.51; 95 % CI 0.32 to 0.81) or begin care in the first trimester (35.0 % vs. 56.5 %; aOR 0.44; 95 % CI 0.29 to 0.66) than non-immigrant-born women (Additional file 2: Table S2 and Additional file 3: Table S3).

**Table 2:** Regression models of predictors of adequate prenatal care (R-GINDEX)

Covariate	Crude Odds Ratio	Adjusted Odds Ratio
	(95 % Confidence Interval)	(95 % Confidence Interval
Women living with HIV	0.74 (0.63 to 0.88)	0.74 (0.62 to 0.88)
Age		
18 to 34 years	1.00	1.00
35 to 49 years	0.72 (0.72 to 0.73)	0.74 (0.74 to 0.75)
Aggregated Diagnosis Groups		
0 to 5	1.00	1.00
6 to 10	1.52 (1.51 to 1.54)	1.53 (1.52 to 1.55)
11 or more	2.16 (2.09 to 2.23)	2.20 (2.12 to 2.27)
Immigration Status		
Non-immigrant	1.00	1.00
Non-recent immigrant, Africa or Caribbean	0.80 (0.78 to 0.83)	0.81 (0.79 to 0.83)
Non-recent immigrant, other world regions	1.05 (1.04 to 1.06)	1.02 (1.00 to 1.03)
Recent immigrant, Africa or Caribbean	0.62 (0.60 to 0.65)	0.65 (0.63 to 0.68)
Recent immigrant, other world regions	0.78 (0.78 to 0.79)	0.81 (0.80 to 0.82)
Material Deprivation Income Quintile		
1 (lowest)	1.00	1.00
2	0.85 (0.84 to 0.86)	0.85 (0.84 to 0.86)
3	0.76 (0.75 to 0.76)	0.76 (0.76 to 0.77)
4	0.69 (0.68 to 0.69)	0.70 (0.69 to 0.71)
5	0.62 (0.61 to 0.63)	0.63 (0.62 to 0.64)
Residential Instability Quintile		
1 (lowest)	1.00	1.00
2	0.98 (0.97 to 0.99)	1.01 (1.00 to 1.02)
3	0.90 (0.89 to 0.91)	0.99 (0.98 to 1.01)
4	0.83 (0.82 to 0.84)	1.00 (0.99 to 1.01)
5	0.83 (0.82 to 0.84)	1.01 (1.00 to 1.02)
Multiple birth	4.12 (3.99 to 4.25)	3.97 (3.84 to 4.10)
Parity	0.87 (0.86 to 0.87)	0.84 (0.83 to 0.85)

**Table 3:** Regression models of initiation of prenatal care in the first trimester

Covariate	Crude Odds Ratio	Adjusted Odds Ratio
	(95 % Confidence Interval)	(95 % Confidence Interval)
Women living with HIV	0.45 (0.38 to 0.53)	0.51 (0.43 to 0.60)
Age		
18 to 34 years	1.00	1.00
35 to 49 years	0.79 (0.78 to 0.80)	0.82 (0.81 to 0.83)
Aggregated Diagnosis Groups		
0 to 5	1.00	1.00
6 to 10	1.43 (1.41 to 1.44)	1.46 (1.45 to 1.47)
11 or more	1.57 (1.51 to 1.63)	1.63 (1.57 to 1.70)
Immigration Status		
Non-immigrant	1.00	1.00
Non-recent immigrant, Africa or Caribbean	0.64 (0.62 to 0.66)	0.67 (0.65 to 0.69)
Non-recent immigrant, other world regions	0.95 (0.93 to 0.96)	0.93 (0.91 to 0.94)
Recent immigrant, Africa or Caribbean	0.46 (0.45 to 0.48)	0.52 (0.50 to 0.53)
Recent immigrant, other world regions	0.64 (0.64 to 0.65)	0.68 (0.67 to 0.69)
Material Deprivation Income Quintile		
1 (lowest)	1.00	1.00
2	0.84 (0.83 to 0.85)	0.85 (0.83 to 0.86)
3	0.73 (0.72 to 0.74)	0.75 (0.74 to 0.76)
4	0.63 (0.63 to 0.64)	0.67 (0.66 to 0.68)
5	0.54 (0.54 to 0.55)	0.59 (0.58 to 0.60)
Residential Instability Quintile		
1 (lowest)	1.00	1.00
2	1.01 (0.99 to 1.02)	1.03 (1.02 to 1.04)
3	0.92 (0.91 to 0.93)	1.02 (1.01 to 1.04)
4	0.80 (0.79 to 0.81)	1.00 (0.99 to 1.02)
5	0.73 (0.72 to 0.74)	0.95 (0.94 to 0.96)
Multiple birth	1.59 (1.54 to 1.64)	1.49 (1.44 to 1.54)
Parity	0.96 (1.54 to 1.64)	0.92 (0.83 to 0.93)

## Discussion

In our population-based study, we found that women living with HIV were less likely to receive adequate prenatal care than women living without HIV. We also found that, among women with HIV, recent immigrants from Africa and the Caribbean were markedly less likely to receive adequate prenatal care than non-immigrant women. Similar findings have been described in other jurisdictions with universal access to prenatal care. Specifically, women with HIV originally from Africa were at heightened risk of late initiation of prenatal care relative to referent populations in separate studies conducted in the UK/Ireland and France, although late diagnosis of HIV accounted for this finding in the latter study [39, 40].

Our findings build on previous research examining the adequacy of prenatal care among women with HIV. In a U.S. study examining 2254 singleton births to women with HIV, only one-third received adequate prenatal care according to a utilization index, with 20 % reporting no prenatal care before delivery [28]. Similar results were noted in a study describing prenatal care utilization by women with HIV in 4 U.S. states, in that 39 % of women did not receive adequate prenatal care [41]. However, these studies were not population-based in nature and were conducted prior to the availability of modern antiretroviral therapy. Moreover, our study was conducted in a setting of universal coverage for prenatal care. Consequently, the receipt of prenatal care should not be influenced by health insurance status.

We speculate that our findings are related to a series of inter-related social and structural barriers to care for women living with HIV during the preconception and prenatal periods. This reasoning is supported by earlier literature documenting stigma, discrimination, difficulty for newcomers navigating a foreign health care system and lack of preconception counselling for women living with HIV, each of which may act as components in one or more causal mechanisms that culminate in the outcome of inadequate prenatal care [17–27].

Our findings have important implications for needs assessment and programme planning. In Canada and other developed countries, great strides have been made in ensuring that the overwhelming majority of women with HIV receive antiretroviral therapy during pregnancy. Consequently, the rate of perinatal HIV transmission among women who receive antenatal is 1 % [42]. However, substantial non-infectious neonatal morbidity exists in the context of HIV infection in Ontario, with risks of low birth weight, preterm birth and small for gestational age births among women with HIV exceeding those of non-infected women by 90 %, 76 % and 43 %, respectively [43]. Because research has shown that improving access to prenatal care is associated with reduced risks of these adverse neonatal outcomes in women with HIV [27], it is important to ensure that additional data are gathered from these women which inform the development of interventions that promote timely linkage to and retention in prenatal care. This may be especially salient for those women originally from Africa and the Caribbean. However, in light of our findings that approximately 40 % of women living with HIV in Ontario who have had children live in the most deprived and unstable neighborhoods in the province, it is unlikely that narrowly targeted interventions which only increase access to prenatal medical services will be sufficient for addressing the social and environmental determinants of perinatal health in these women [44–46]. Many women living with HIV who are pregnant may also have immigration, housing, legal and social support needs which must also be addressed. Further qualitative research is planned with the community of women living with HIV to evaluate and explore the nature of prenatal services required by women with HIV.

Several limitations of our study merit emphasis. First, as with other utilization indices, the R-GINDEX is a quantitative measure of prenatal care use and does not address the quality or content of care received. Second, prenatal care provided through midwives, community support programs, nurses or physicians who do not bill OHIP is not recorded in our administrative databases. It is therefore possible that the adequacy of prenatal care was under-estimated in our study, particularly among women originally from Africa and the Caribbean. Third, we could not ascertain births among women who were refugee claimants or who did not have provincial health insurance. Fourth, the R-GINDEX is based on the American College of Obstetricians and Gynecologists recommendations for the number of visits for low risk pregnant women. The applicability of this measure to women living with HIV is unknown. Finally, our databases did not include information about other determinants of prenatal health use including substance use, maternal education and intendedness of pregnancy [26, 35].

## Conclusions

We identified meaningful disparities in the receipt of adequate prenatal care between women with and without HIV and among women with HIV originally from Africa and the Caribbean relative to Canadian-born women. Because understanding and addressing barriers to care is complex and multifaceted, involving women with HIV in research and policy initiatives which facilitate the use of prenatal care and characterize the content of prenatal care required to address the social determinants of pregnancy outcomes is warranted.

## Additional files

Additional file 1: Table S1. R-GINDEX prenatal care categories for 28-, 32-, 36- and 40-week deliveries. Additional file 2: Table S2. Demographic characteristics of pregnancies among women with HIV, by immigration status. Additional file 3: Table S3. Multivariable analyses of predictors of adequate prenatal care and initiation of prenatal care in first trimester among women with HIV.

## Abbreviations

HIV: Human immunodeficiency virus
OHIP: Ontario health insurance plan
R-GINDEX: Revised-graduated prenatal care utilization index
aOR: Adjusted odds ratio
CI: Confidence interval
