# Supplementary material for: Adequacy of prenatal care among women living with human immunodeficiency virus: a population-based study
Source: BMC Public Health. 2015 May 29;15:514. doi: 10.1186/s12889-015-1842-y (PMC4462120; doi:10.1186/s12889-015-1842-y)
Supplement: Additional file 1: Table S1. — R-GINDEX prenatal care categories for 28-, 32-, 36- and 40-week deliveries. [file 12889_2015_1842_MOESM1_ESM.doc]

Additional file 1: Table S1: R-GINDEX prenatal care categories for 28-, 32-, 36- and 40-week deliveries

| **R-GINDEX Category** | **28-week delivery** | | **32-week delivery** | | **36-week delivery** | | **40-week delivery** | |
| --- | --- | --- | --- | --- | --- | --- | --- | --- |
|  | Months PNC began | Number of PNC visits | Months PNC began | Number of PNC visits | Months PNC began | Number of PNC visits | Months PNC began | Number of PNC visits |
| Intensive | 1 to 3 | > 14 | 1 to 3 | > 16 | 1 to 3 | > 16 | 1 to 3 | > 17 |
|  | 4 to 6 | > 12 | 4 to 6 | > 13 | 4 to 6 | > 14 | 4 to 6 | > 15 |
|  | > 7 | > 10 | > 7 | > 11 | > 7 | > 12 | > 7 | > 13 |
| Adequate | 1 to 3 | 5 to 13 | 1 to 3 | 7 to 15 | 1 to 3 | 9 to 15 | 1 to 3 | 13 to 16 |
| Intermediate | 1 to 3 | 2 to 4 | 1 to 3 | 4 to 6 | 1 to 3 | 5 to 8 | 1 to 3 | 8 to 12 |
|  | 4 to 6 | 2 to 11 | 4 to 6 | 4 to 12 | 4 to 6 | 6 to 13 | 4 to 6 | 8 to 14 |
| Inadequate | 1 to 3 | 1 | 1 to 3 | 1 to 3 | 1 to 3 | 1 to 4 | 1 to 3 | 1 to 7 |
|  | 4 to 6 | 1 | 4 to 6 | 1 to 3 | 4 to 6 | 1 to 5 | 4 to 6 | 1 to 7 |
|  | > 7 | 1 to 9 | > 7 | 1 to 10 | > 7 | 1 to 11 | > 7 | 1 to 12 |
| No Care |  | 0 |  | 0 |  | 0 |  | 0 |

PNC, prenatal care
