# Supplementary material for: Adequacy of prenatal care among women living with human immunodeficiency virus: a population-based study
Source: BMC Public Health. 2015 May 29;15:514. doi: 10.1186/s12889-015-1842-y (PMC4462120; doi:10.1186/s12889-015-1842-y)
Supplement: Additional file 2: Table S2. — Demographic characteristics of pregnancies among women with HIV, by immigration status. [file 12889_2015_1842_MOESM2_ESM.doc]

Additional file 2: Table S2: Demographic characteristics of pregnancies among women with HIV, by immigration status

| **Characteristic** | **Immigrant**  **(n = 305)** | **Non-immigrant**  **(n = 329)** | **p-value** |
| --- | --- | --- | --- |
| Mean age + SD (years) | 31.4 + 5.1 | 30.2 + 5.3 | 0.004 |
| 18 to 34 years | 216 (70.8%) | 254 (77.2%) | 0.07 |
| 35 to 49 years | 89 (29.2%) | 75 (22.8%) |  |
| Aggregated Diagnosis Groups |  |  |  |
| Median (IQR) | 7.0 (5.0 – 8.0) | 6.0 (4.0 – 9.0) | 0.47 |
| 0 to 5 | 106 (34.8%) | 132 (40.1%) | 0.12 |
| 6 to 10 | 172 (56.4%) | 159 (48.3%) |  |
| 11 or more | 27 (8.9%) | 38 (11.6%) |  |
| Material Deprivation Income Quintile, No. (%) |  |  | < 0.001 |
| 1 (lowest) | 24 (7.9%) | 44 (13.4%) |  |
| 2 | 22 (7.2%) | 50 (15.2%) |  |
| 3 | 42 (13.8%) | 56 (17.0%) |  |
| 4 | 51 (16.7%) | 66 (20.1%) |  |
| 5 | 158 (51.8%) | 103 (31.3%) |  |
| Residential Instability Quintile, No. (%) |  |  | 0.05 |
| 1 (lowest) | 33 (10.8%) | 44 (13.4%) |  |
| 2 | 28 (9.2%) | 44 (13.4%) |  |
| 3 | 25 (8.2%) | 44 (13.4%) |  |
| 4 | 79 (25.9%) | 66 (20.1%) |  |
| 5 | 132 (43.3%) | 121 (36.8%) |  |
| Median (IQR) gestational age (weeks) | 38 (37 – 40) | 38 (37 – 40) | 0.67 |

SD, standard deviation; IQR, interquartile range
