# Supplementary material for: Adequacy of prenatal care among women living with human immunodeficiency virus: a population-based study
Source: BMC Public Health. 2015 May 29;15:514. doi: 10.1186/s12889-015-1842-y (PMC4462120; doi:10.1186/s12889-015-1842-y)
Supplement: Additional file 3: Table S3. — Multivariable analyses of predictors of adequate prenatal care and initiation of prenatal care in first trimester among women with HIV. [file 12889_2015_1842_MOESM3_ESM.doc]

Additional file 3: Table S3: Multivariable analyses of predictors of adequate prenatal care and initiation of prenatal care in first trimester among women with HIV

| **Covariate** | **Adequacy of prenatal care (adjusted odds ratio and 95% CI)** | **Initiation of prenatal care in first trimester (adjusted odds ratio and 95% CI)** |
| --- | --- | --- |
| Age |  |  |
| 18 to 34 years | 1.00 | 1.00 |
| 35 to 49 years | 1.06 (0.72 to 1.58) | 0.90 (0.61 to 1.31) |
| Aggregated Diagnosis Groups |  |  |
| 0 to 5 | 1.00 | 1.00 |
| 6 to 10 | 2.93 (1.97 to 4.37) | 1.98 (1.39 to 2.83) |
| 11 or more | 2.83 (1.58 to 5.06) | 1.79 (1.03 to 3.12) |
| Immigration Status |  |  |
| Non-immigrant | 1.00 | 1.00 |
| Non-recent immigrant, Africa or Caribbean | 0.88 (0.52 to 1.50) | 1.02 (0.62 to 1.69) |
| Non-recent immigrant, other world regions | 1.16 (0.52 to 2.59) | 0.50 (0.22 to 1.12) |
| Recent immigrant, Africa or Caribbean | 0.51 (0.32 to 0.81) | 0.44 (0.29 to 0.66) |
| Recent immigrant, other world regions | 0.66 (0.26 to 1.71) | 0.74 (0.30 to 1.80) |
| Material Deprivation Income Quintile, No. (%) |  |  |
| 1 (lowest) | 1.00 | 1.00 |
| 2 | 1.53 (0.72 to 3.25) | 1.15 (0.59 to 2.23) |
| 3 | 1.62 (0.83 to 3.15) | 1.51 (0.79 to 2.88) |
| 4 | 1.26 (0.64 to 2.49) | 0.98 (0.52 to 1.84) |
| 5 | 1.68 (0.90 to 3.14) | 0.97 (0.54 to 1.73) |
| Residential Instability Quintile, No. (%) |  |  |
| 1 (lowest) | 1.00 | 1.00 |
| 2 | 0.91 (0.47 to 1.77) | 1.07 (0.54 to 2.13) |
| 3 | 0.56 (0.28 to 1.12) | 0.80 (0.41 to 1.57) |
| 4 | 0.51 (0.27 to 0.96) | 1.08 (0.56 to 2.10) |
| 5 | 0.40 (0.21 to 0.74) | 0.64 (0.34 to 1.19) |
| Multiple birth | 1.18 (0.48 to 2.91) | 1.40 (0.52 to 3.75) |
| Parity | 0.64 (0.43 to 0.94) | 0.89 (0.62 to 1.28) |
